# Correcting Congenital Talipes Equinovarus in Children Using Three Different Corrective Methods: A Consort Study

**DOI:** 10.1097/MD.0000000000001004

**Published:** 2015-07-17

**Authors:** Wei Chen, Fang Pu, Yang Yang, Jie Yao, Lizhen Wang, Hong Liu, Yubo Fan

**Affiliations:** From the Key Laboratory of Rehabilitation Technical Aids, Ministry of Civil Affair, School of Biological Science and Medical Engineering, Beihang University (WC, FP, YY, JY, LW, YF); State Key Laboratory of Virtual Reality Technology and Systems, Beihang University (FP, YF); National Research Center for Rehabilitation Technical Aids (YF); and Rokab Pedorthic Center, Beijing, P. R. China (HL).

## Abstract

Equinus, varus, cavus, and adduction are typical signs of congenital talipes equinovarus (CTEV). Forefoot adduction remains a difficulty from using previous corrective methods. This study aims to develop a corrective method to reduce the severity of forefoot adduction of CTEV children with moderate deformities during their walking age. The devised method was compared with 2 other common corrective methods to evaluate its effectiveness.

A Dennis Brown (DB) splint, DB splint with orthopedic shoes (OS), and forefoot abduct shoes (FAS) with OS were, respectively, applied to 15, 20, and 18 CTEV children with moderate deformities who were scored at their first visit according to the Diméglio classification. The mean follow-up was 44 months and the orthoses were changed as the children grew. A 3D scanner and a high-resolution pedobarograph were used to record morphological characteristics and plantar pressure distribution. One-way MAVONA analysis was used to compare the bimalleolar angle, bean–shape ratio, and pressure ratios in each study group.

There were significant differences in the FAS+OS group compared to the DB and DB+OS groups (*P* < 0.05) for most measurements. The most salient differences were as follows: the FAS+OS group had a significantly greater bimalleolar angle (*P* < 0.05) and lower bean–shape ratio (*P* < 0.01) than the other groups; the DB+OS and FAS+OS groups had higher heel/forefoot and heel/LMF ratios (*P* < 0.01 and *P* < 0.001) than the DB group.

FAS are critical for correcting improper forefoot adduction and OS are important for the correction of equinus and varus in moderately afflicted CTEV children. This study suggests that the use of FAS+OS may improve treatment outcomes for moderate CTEV children who do not show signs of serious torsional deformity.

## INTRODUCTION

Congenital talipes equinovarus (CTEV), or clubfoot, is a common foot deformity that involves a complex three-dimensional musculoskeletal abnormality.^1^ The deformity has 4 main components: equinus, varus, cavus, and adduction.^1,2^ If the deformity is not corrected promptly, the ambulatory ability of children will be seriously affected. Nonoperative treatments are typically considered the first choice for treating CTEV in young children.^3^

During the prewalking period, the Ponseti method is usually regarded as the standard initial treatment for CTEV.^1,4–6^ For short-term effect of the Ponseti treatment, corrective bracing is used following initial correction.^7^ According to published reports,^8^ the highest rate of recurrence occurs between 1.5 and 4 years of age. Therefore, correction is still needed even for children with CTEV who are beginning to walk.

Foot abduction braces are typically used for continuous correction.^7^ Ponseti and Smoley^9^ reported an early analysis of a Denis Browne (DB) splint, which is a commonly used corrective device. This brace consists of open-toed, high-top, straight-lace shoes attached in external rotation to a bar,^10^ and is used to hold the affected foot at approximately 70° and the unaffected foot at approximately 40° of external rotation.^7^ DB splints must be worn every night for 2–4 years.^7,11,12^ Some studies concluded that the DB splint could correct equinus and varus, but residual adduction of the foot would still exist.^3,13^ Furthermore, given that the shoes are attached to a bar, there is a high rate of noncompliance due to difficulties with use for years on end.^4^

Orthopedic shoes (OS) are considered more convenient than DB splints and can be used for walking.^14^ Moreover, weight-bearing is also important for effectively correcting CTEV. OS are custom-made shoes with inserts that are molded to the shape of the hind foot so as to hold it firmly. OS have been reported with positive results for correcting equinus and varus in weight-bearing correction.^15,16^ However, considering that OS are usually set in a neutral alignment and fail to provide abduction correction, they could not be used to stretch medial structures,^17^ and residual adduction is also present after treatment.^18^

Reiman^19^ proposed a dynamic splint to correct adduction. An elastic cord on the splint's lateral side acts to support the cuboid and exert the necessary counter-pressure for abduction of the forefoot. However, considering its complexity and difficulty with use, the splint can only be used at night, and is thus not commonly used in clinical practice.

This paper introduces a corrective method that consists of the daytime and nighttime use of orthoses. Orthopedic shoes are used during the daytime, while forefoot abduction shoes are used at night. This paper will also explore the outcome of this new corrective method in comparison to 2 other common corrective methods for children with CTEV.

## METHOD

### Corrective Treatment Methods

**FIGURE 1:**
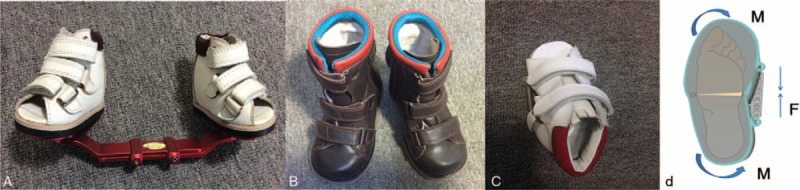
From left to right: A, Dennis Brown Splint; B, orthopedic shoe with orthopedic insole in it. There are anterior and posterior outflares on the shoes and a velcro in the malleolar area to control equinus; C, top view of forefoot abduct shoe for left foot; and D, the schematic bottom view of the forefoot abduct shoe for left foot.

DB splints comprise a pair of boots connected by a rigid bar, as shown in Figure 1A. A kind of forefoot abduction shoe (FAS) specially designed to correct adduction deformities is shown in Figure 1C. Abduction of the forefoot was controlled by a spring placed on the lateral side of the FAS, as shown in Figure 1D. The FAS is a 2-piece orthosis with an adjustable spring between the lateral hindfoot and lateral forefoot. An ankle-fixing strap is placed inside the shoe to secure the heel. The shoe was initially set with a 20° to 25° outflare according to the principle of the Bēbax shoe.^27^ When the spring contracts, the FAS retains the outflare, and the foot remains in abduction. For unilateral children the unaffected foot is free and for bilateral children a pair of FAS was needed at night. Given that the DB and FAS are only used at night, orthopedic shoes (OS) were used while walking, as shown in Figure 1B. The OS were used with an orthopedic insole, and the hard heel cup and upper shoe helps to keep the heel in a neutral position. To evaluate the function of different orthoses, these 3 different corrective methods were evaluated on children who were in the early stages of walking: DB, DB+OS, and FAS+OS.

### Subjects

This is a prospective, single-blinded, randomized, controlled trial. A total of 113 children with CTEV were recruited after Ponseti treatment. All children were participating following successful initial management using the Ponseti method and had been braced with a DB splint during their prewalking stages. The feet were examined and scored at the first visit according to the Diméglio classification,^20^ which has been considered the most reliable classification method.^21^ There was no significant difference in equinus, varus, and addutus deformities between them based on the scores (*P* > 0.05). After the Diméglio classification, 53 children with moderate deformities were recruited. All the children in this study had finished Ponseti treatment and were wearing DB splints for the initial period of correction, which effectively controlled torsional deformity. When they began to walk, 15 children continued to use a DB splint at night. A total of 20 children used DB splint for nighttime use and OS for daytime use. A total of 18 children accepted FAS for nighttime use and OS for daytime use. For unilateral children, the unaffected foot was not restricted or constrained. The data were collected when the children were between 4–5 years of age. Informed consent was obtained from their parents. Follow-up visits were done, and the orthoses were changed as the children grew. The mean follow-up time was 44 months. Table 1 shows the demographics of the children after they underwent corrective treatment. No significant differences were found in age, height, and weight among the three groups (*P* > 0.05). Informed written consent was obtained from the parents of each subject in accordance with clinical protocols. This study was approved by the Science and Ethics Committee of School of Biological Science and Medical Engineering at Beihang University, Beijing, China, on November 9, 2010 (Approval ID: 20101109).

**TABLE 1:**
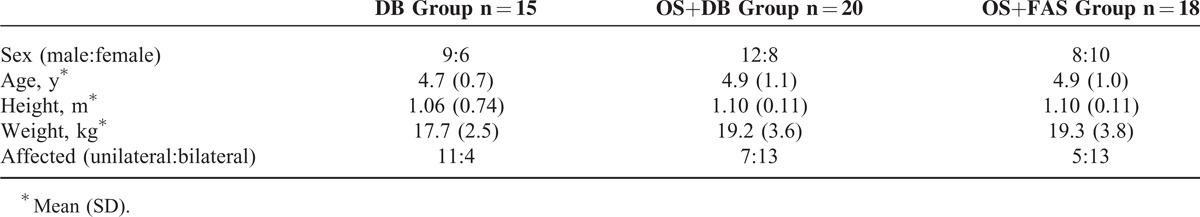
Demographic Characteristics of the Participants of This Study

### Procedure

All participants underwent three-dimensional foot scanning and pedobarography. All data were captured by an experienced pedorthic doctor and a bioengineer in the gait analysis laboratory at Rokab Pedorthic Center, Beijing, China.

**FIGURE 2:**
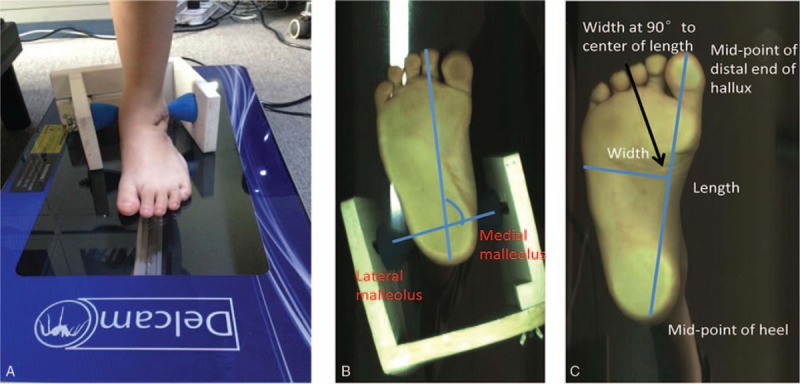
A, 3D scanner and malleolar jig for measuring bimalleolar angle. B, The bimalleolar angle is formed between the bimalleolar axis and the longitudinal axis of the foot passing through the second toe. C, Bean–shape ratio is foot width-to-length ratio.

A Delcam Iqube scanner (Delcam, Birmingham, UK) and a malleolar jig were used to collect and calculate the malleolar angles (Figure 2A). The malleolar angle is determined from a scanned image of the plantar aspect of the foot while standing still (Figure 2B). The malleolar jig was adjusted to fit and was aligned with the transmalleolar axis of each subject, and the medial and lateral malleoli were registered to the scanned image of the foot.^22^ From this image, the bean–shape ratio (Figure 2C), which was calculated by width to length,^24^ could be obtained for each feet.

**FIGURE 3:**
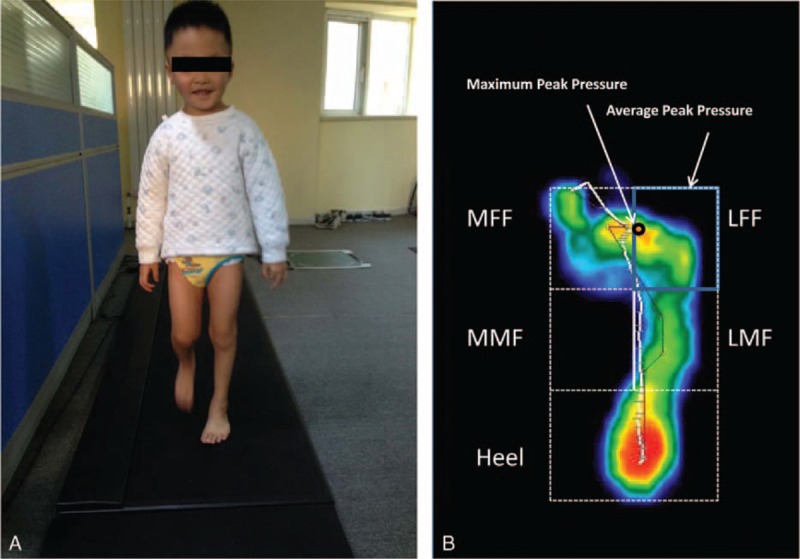
A, Pedobarography for static and dynamic measurements; B, the 5 segments of the foot from pressure image. LFF = lateral forefoot; LMF = lateral midfoot; MFF = medial forefoot; MMF = medial midfoot.

A FreeMed^®^ baropodometric platform, as shown in Figure 3A, was used to measure the dynamic foot pressure (Rome, Italy). The platform surface was 240 × 50 cm, with an active surface of 244 × 74 cm and thickness of 0.8 cm (Sensormédica^®^, Italy). The reliability of this baropodometric platform has been shown in previous studies.^23^ Dynamic measurements required the children to walk at their natural self-selected speed. Four recordings of each affected foot were taken, and the average was considered the final measurement. The FreeStep system^®^ was used to provide information during gait.

### Data Processing

The bimalleolar angle and bean–shape ratio were used to quantify the level of forefoot adduction.^2,24,25^ The bimalleolar angle is the anteromedial angle which is formed between the bimalleolar axis and the longitudinal axis of the foot passing through the second toe. The bean–shape ratio assesses the curvature of the foot, thus quantifying forefoot adduction and hindfoot varus. The average and maximum peak pressure on each region (Figure 3B) were calculated according to methods detailed in the literature.^26^ The MFF/LFF ratio, heel/forefoot ratio, and heel/LMF ratio were calculated to evaluate the degree of equinus and varus deformity in CTEV children.^24,26^

### Statistical Analysis

Statistical analyses were performed using the SPSS for Windows version 19.0 (IBM Corp, Armonk, NY, USA). Continuous variables were reported as means ± SD. The unaffected foot was not used for calculation during statistical analysis. One-way MAVONA analysis was used to evaluate the effects of different corrective methods on the bimalleolar angle, bean–shape ratio, and pressure distribution. Least-significant-difference tests were used for posthoc comparisons. Power analysis was performed using PASS software (Jerry Hintze, Kaysville. UT). The significance level was set at 0.05.

## RESULTS

A total of 53 children were included in this study. There were no significant differences in varus, equines, and adduction deformities between the 3 groups according to the Diméglio score (*P* > 0.05) at initial inception. A total of 15, 20, and 18 children comprised the groups DB, DB+OS, and FAS+OS, respectively. The study began in 2010 and the mean follow-up time was 44 months. No patients withdrew from the treatment during the study period. The FAS+OS group showed better compliance than the other 2 groups, as reported by the children's parents. Although physiotherapy and orthoses were continuous, 5 children in the DB group and 1 child in the DB+OS group exhibited severe equinus, varus, and adduction deformities after treatment. Surgery may still be needed to correct these severe deformities.

MANOVA revealed that the different corrective methods had a primary influence on the bimalleolar angle and bean–shape ratio, as shown in Table 2. For the 3 groups, DB, DB+OS, and FAS+OS, the bimalleolar angle was found to successively increase [*F*_(2,80)_ = 3.598, *P* = 0.032] and bean–shape ratio to successively decrease [*F*_(2,80)_ = 6.852, *P* = 0.002], respectively. A significant difference in bimalleolar angle was found between the DB and FAS+OS groups (posthoc comparison: *P* = 0.009). Also, a significant difference in bean–shape ratio was found between the DB and DB+OS groups (posthoc comparison: *P* = 0.049), as well as between the DB+OS and FAS+OS groups (posthoc comparison: *P* < 0.001).

**TABLE 2:**
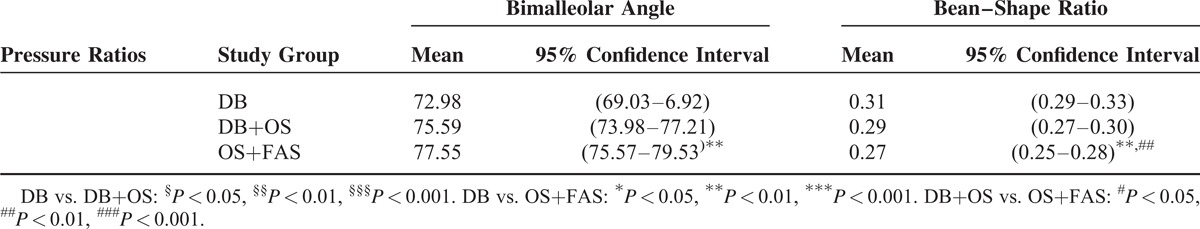
Abduction Indexes Adapted From Ramanathan et al^25^

It was also found that different corrective methods had a significant impact on the peak pressure at the hindfoot, LMF, and MFF; MANOVA results are shown in Table 3. For the DB, DB+OS, and FAS+OS groups, both the maximum and average peak pressures successively increased at the heel [*F*_(2,80)_ = 12.664, *P* < 0.001 and *F*_(2,80)_ = 3.896, *P* = 0.024]. However, both the maximum and average peak pressures successively decreased at the lateral midfoot [*F*_(2,80)_ = 3.564, *P* = 0.033 and *F*_(2,80)_ = 5.761, *P* = 0.005], and both the maximum and average peak pressures successively increased in the medial forefoot [*F*_(2,80)_ = 5.178, *P* = 0.008 and *F*_(2,80)_ = 2.892, *P* = 0.049].

**TABLE 3:**
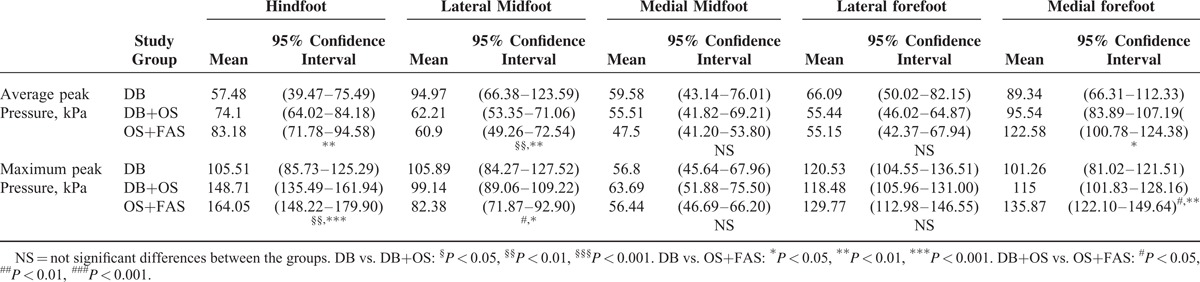
Results for Pressure and Forces Expressed as the Estimated Mean and the 95% Confidence Intervals From the Linear Mixed Model Analysis

As shown in Table 4, MANOVA revealed that different corrective methods had a primary influence on the peak pressure ratios of heel/forefoot and heel/LMF. For the DB, DB+OS, and FAS+OS groups, both the heel/forefoot and heel/LMF ratio successively increased [*F*_(2,80)_ = 4.975, *P* = 0.009 and *F*_(2,80)_ = 14.878, *P* < 0.001]. A significant difference in heel/forefoot ratio was found between the DB and DB+OS groups (posthoc comparison: *P* = 0.006), as well as between the DB and FAS+OS groups (posthoc comparison: *P* = 0.005). A significant difference in the heel/lateral arch ratio was found among the DB, DB+OS, DB+OS groups (posthoc comparison: *P* = 0.003, *P* < 0.001, *P* = 0.007). For the significantly different variables (bimalleolar angle, bean–shape ratio, heel/forefoot, and heel/LMF), the power ranged from 0.65 to 0.99.

**TABLE 4:**
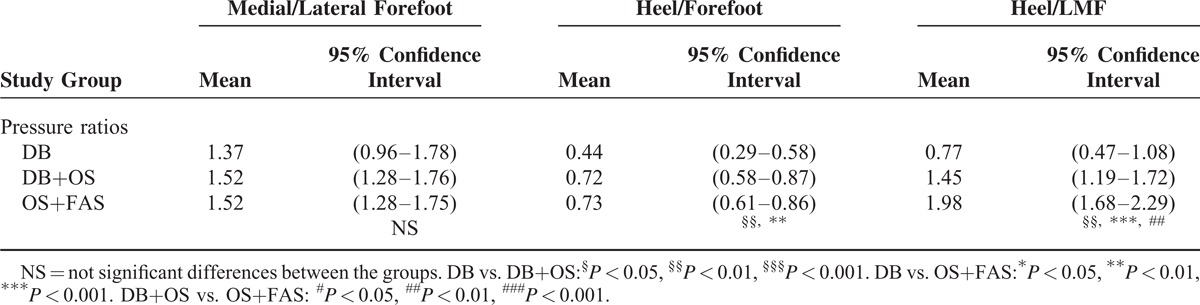
Peak Pressure Ratios Adapted From Herd et al

## DISCUSSION

When treating CTEV, the corrective method used, or whether correction was used at all, will have a great impact on patient outcome. This article introduces a novel corrective method for CTEV that combines the daytime use of orthopedic shoes (OS) and nighttime use of forefoot abduction shoes (FAS). The outcome of this new approach was studied in comparison with the use of a Dennis Brown (DB) splint and a combination of DB+OS.

Our results demonstrate that the FAS+OS group exhibits superior correction of abnormal adduction (has the greatest bimalloeolar angle of 77.55° and the lowest bean–shape ratio of 0.27). A bimalleolar angle of less than 80° is considered abnormal.^25^ Although the use of FAS+OS resulted in an angle of less than 80°, it achieved a higher value than the other 2 groups and a significant difference was observed between the FAS+OS and the DB groups (posthoc comparison: *P* = 0.009). The bean–shape ratio is a comprehensive index that indicates forefoot adduction as well as hindfoot varus.^3^ The more curved the foot in the transverse plane, the higher the ratios were. If values are greater than 0.267, a bean-shaped foot is indicated, and if the value is greater than 0.34, a marked deformity is denoted.^24^ The bean–shape ratio in the FAS+OS group was 0.27, which is the closest to the normal value of 0.23 ± 0.02. A significantly greater bimalloeolar angle and lower bean–shape ratio was observed in the FAS+OS group compared with the DB and DB+OS groups (posthoc comparison: *P* < 0.001, *P* = 0.049). The result demonstrates that FAS may be critical to correcting forefoot adduction, and OS+FAS has a positive effect in correcting forefoot adduction and varus. Some studies have proposed that if the medial soft tissue is released, the release of the medial plantar fascia, tendon, and ligament can help to correct varus.^28^ The bean–shape ratio has been considered a sensitive indicator of relapse.^24^ Our results suggest that FAS+OS placed the foot into a position with a low bean–shape ratio, which could help decrease adduction relapse in patients with CTEV.

Our results also demonstrate that OS have a positive effect on the correction of equino-varus deformities in the OS and FAS+OS groups. These results are reflected by the heel/forefoot and heel/ LMF ratios. If the value of heel/forefoot ratio is less than 0.8, an equinus deformity is considered. If the value is less than 0.4, severe equinus deformity is considered.^25^ The average values of heel/forefoot ratios of the DB and DB+OS groups were 0.44 and 0.72, respectively. A significant difference was observed in the DB+OS group when compared with the DB group (posthoc comparison: *P* = 0.006). The results demonstrated that OS is critical to the correcting equinus in CETV. The heel/LMF ratio is considered a sensitive indicator of equino-varus deformity.^24^ If the ratio is less than 2.5, an equino-varus deformity is typically diagnosed. If the ratio is less than 1.0, a severe equino-varus deformity is considered.^25^ The average values of the heel/LMF ratios of the DB and DB+OS groups were 0.77 and 1.45, respectively. A significant difference was observed among the DB and DB+OS groups (posthoc comparison: *P* = 0.003, and *P* = 0.007). The results also demonstrate that OS have a positive effect on the correction of equino-varus deformities in CETV.

Compared with previous research,^26^ this study found lower values for both heel/forefoot and heel/LMF pressure ratio in the DB group. This difference may be attributed to the younger age of the children in our study, and their noncompliance to the DB splint. Noncompliance may be considered to be the greatest barrier to successful DB splinting.^3,12^ A lack of adherence to the bracing protocol increases the risk of relapse. In this study, most DB group parents found it difficult to persist in donning the DB splint every night, and this may be why the DB group in our study exhibited more evident equinus and varus deformities. Better compliance was observed in FAS+OS group. Most CTEV children could persist wearing these 2 kinds of orthoses every day and night, and their parents were satisfied with the new corrective methods.

This work presents essential information on foot morphological characteristics and pressure characteristics in children with CTEV treated with different corrective techniques. This quantitative description is not only objective but also reproducible, and could provide more useful parameters than single recurrence rates or clinical evaluation. However, this study has some limitations:

The corrective methods used do not consider severe foot deformities. Also, FAS is used for controlling adduction, it is not effective for controlling other types of deformity. FAS+OS design applies on those children who do not have serious torsional deformity.

The bias of the patients recruited in the study is extremely wide, even though it is a randomized study. There are many other conditions such as muscle strength, cooperation with physiotherapy, and cognitive skills which may have an effect on the results.

## CONCLUSION

This article developed a simple manner of correction that consists of the daytime and nighttime use of orthoses for moderate CTEV children. OS used during the day mainly control equinus and varus, while FAS are used at night to correct forefoot adduction. This novel method achieves better corrective results for controlling equinovarus, especially in forefoot adduction, in comparison to other conventional treatment regimes. Compliance is also improved. Therefore, this approach may be a more appropriate treatment option for CTEV children with moderate foot deformities and without serious torsional deformity.

## References

[1] Yapp LZ Arnold GP Nasir S Assessment of talipes equinovarus treated by Ponseti technique: three-year preliminary report. *Foot* 2012; 22:90–94. 10.1016/j.foot.2012.01.001 22387138

[2] Liao HF Cai AL Bing W Value of the fetal plantar shape in prenatal diagnosis of talipes equinovarus. *JUM* 2012; 31:997–1002. 10.7863/jum.2012.31.7.997 22733848

[3] Parsa A Moghadam MH Mohammad HJ Relapsing and residual clubfoot deformities after the application of the Ponseti method: a contemporary review. *Arch Bone Jt Surg* 2014; 2:7–10. 25207306 PMC4151443

[4] Lourenço AF Morcuende JA Correction of neglected idiopathic club foot by the Ponseti method. *J Bone Joint Surg Br* 2007; 89:378–381. 17356154 10.1302/0301-620X.89B3.18313

[5] Colburn M Williams M Evaluation of the treatment of idiopathic clubfoot by using the Ponseti method. *J Foot Ankle Surg* 2003; 42:259–267. 14566717 10.1016/s1067-2516(03)00312-0

[6] Geoffrey FH Cameron GW Haemish A Early clubfoot recurrence after use of the Ponseti method in a New Zealand population. *J Bone Joint Surg Am* 2007; 89:487–493. 17332096 10.2106/JBJS.F.00169

[7] Zionts LE Frederick RD Bracing following correction of idiopathic clubfoot using the Ponseti method. *J Am Acad Orthop Surg* 2010; 18:486–493. 20675641 10.5435/00124635-201008000-00005

[8] Ippolito E Farsetti P Valentini MB Bentley G Management of clubfoot. *European Surgical Orthopaedics Traumatology* 2014 4483–4510.DOI 10.1007/978-3-642-34746-7_157.

[9] Smoley EN Ponseti IV Congenital club foot: the results of treatment. *J Bone Joint Surg Am* 1963; 45:261–275.

[10] Chen RC Gordon JE Luhmann SJ A new dynamic foot abduction orthosis for clubfoot treatment. *J Pediatr Orthopaedics* 2007; 27:522–528. 10.1097/bpo.0b013e318070cc19 17585260

[11] Ponseti IV Congenital Clubfoot, in Fundamentals of Treatment. New York: Oxford University Press; 1996.

[12] Paschoal MM Nogueira M Kathleen F The Ponseti method of treatment for clubfoot in Brazil: barriers to bracing compliance. *Iowa Orthop* 2013; 33:161–166. PMC3748873 24027477

[13] Zahid Z Zia M Awan M The rate of recurrence of club foot deformity in patients using Dennis brown splint. *IJRS* 2012; 1:58–62.

[14] Janicki JA Wright JG Weir S A comparison of ankle foot orthoses with foot abduction orthoses to prevent recurrence following correction of idiopathic clubfoot by the Ponseti method. *J Bone Joint Surg Br* 2011; 93:700–704. 21511939 10.1302/0301-620X.93B5.24883

[15] Rizza R Liu XC Thometz J A new method in the design of a dynamic pedorthosis for children with residual clubfoot. *J Med Devices* 2010; 4:1–5.

[16] Jain ML Dhande SG Vyas NS Virtual modeling of an ankle foot orthosis for correction of foot abnormality. *Robot Computer-Integr* 2011; 27:257–260.

[17] Desai L Oprescu F Dimeo A Bracing in the treatment of children with clubfoot past, present, and future. *Iowa Orthop J* 2010; 30:15–23. 21045966 PMC2958265

[18] Heilig MR Matern RV Rosenzweig SD Current management of idiopathic clubfoot questionnaire: a multicentric study. *J Pediatr Orthopaedics* 2003; 23:780–787. 10.1097/00004694-200311000-00017 14581783

[19] Reimann I Lyquist E Dynamic splint used in the treatment of club foot. *Acta Orthop Scand* 1969; 40:817–824. 5376792 10.3109/17453676908989546

[20] Diméglio A Bensahel H Souchet P Classification of clubfoot. *J Pediatr Orthop B* 1995; 4:129–136. 7670979 10.1097/01202412-199504020-00002

[21] Wainwright AM Auld T Benson MK The classification of congenital talipes equinovarus. *J Bone Joint Surg Br* 2002; 84:1020–1024. 12358365 10.1302/0301-620x.84b7.12909

[22] Lamm BM Mendicino RW Catanzariti AR Static rearfoot alignment: a comparison of clinical and radiographic measures. *J Am Podiatr Med Assoc* 2005; 95:26–33. 15659411 10.7547/0950026

[23] Romero-franco N Martínez-amat A Martínez-lópez EJ Short-term effects of a proprioceptive training session with unstable platforms on the monopodal stabilometry of athletes. *J Phys Therapy Sci* 2014; 26:45–51. 10.1589/jpts.26.45 PMC3927040 24567674

[24] Herd F Ramanathan AK Cochrane LA Foot pressure in clubfoot: the development of an objective assessment tool. *Foot* 2008; 18:99–105. 10.1016/j.foot.2008.01.007 20307419

[25] Ramanathan AK Abboud RJ Clubfoot assessment the complete IMAR footprint. *Orthopaedics Trauma* 2010; 24:303–308.

[26] Salazar-torres JJ Mcdowell BC Humphreys LD Plantar pressures in children with congenital talipes equino varus: a comparison between surgical management and the Ponseti technique. *Gait Postur* 2014; 39:321–327. 10.1016/j.gaitpost.2013.07.119 23973353

[27] Allen WD Weiner DS Riley PM The treatment of rigid metatarsus adductovarus with the use of a new hinged adjustable shoe orthosis. *Foot Ankle Int* 1993; 14:450–454. 10.1177/107110079301400804 8253437

[28] Mchale KA Lenhart MK Treatment of residual clubfoot Deformity-the Bean-Shaped Foot-by opening wedge medial cuneiform osteotomy and closing wedge cuboid osteotomy: clinical review and cadaver correlations. *J Pediatr Orthopaedics* 1991; 11:374–381. 2056088

